# Causes of Diseases

**Published:** 1881-04

**Authors:** 


HALL'S

### JOURNAL OF HEALTH

AND

MISCELLANY.

E. H. GIBBS, A. M., M. D. - - EDITOR.

Founded in 1854, and published Monthly without Interruption since that Time.

### CAUSES OF DISEASE.

WHATEVER interferes with the regular action of the vital organs produces disease. Perfect health consists in the harmonious working together of the complicated machinery of the system; that we call life.

We are constantly beset with so many dangers, and overtaken by so many vicissitudes, that but few persons of adult age have sound health. Thus a majority of all who are now living are doomed to die—from causes entirely preventable—at ages averaging thirty years less than they should have attained. It is a source of great satisfaction to those who have contributed to the result, to know that mainly through their efforts the average length of human life in this country has been increased by ten years during the last half century. If the time ever arrives when all intelligent men and women shall be familiar with the laws of health, the best methods of meeting accidents and emergencies, and the use of simple remedies on the approach of disease, thirty years more will be added to this average term, and a vast aggregate of suffering saved. These are not the only advantages to be gained by a general dissemination of such knowledge. An improved condition of the general health of the people would add vigor to all the affairs of life. At present the professions and the great business interests of the country are largely represented by men who are invalids, and bear their burdens wearily.

Of the causes of disease the first is by inheritance. Physicians have modified their views in respect of such diseases, and we now say that a person inherits *a tendency* to certain diseases; that is, he is born with a weakness, which, through neglect, might develop disease. The most common, and perhaps the most unfortunate, inheritance is drunkenness; it requires careful attention during childhood.

Constipation is undoubtedly the commencement of more fatal diseases than any other cause. It frequently begins in infancy, and then may be easily and effectually cured; but few parents seem to be aware of the importance of attention to their children in this respect, and in a few years they may become confirmed invalids from this cause alone. All malarial diseases, most types of fever, and ordinary rheumatic affections are due to a torpid condition of the liver, which may be remedied, generally in twenty-four hours, by proper treatment; still thousands of lives are yearly sacrificed by these diseases, through ignorance or neglect. Thousands of other lives are lost by diseases that commenced by taking cold; yet a mild cathartic, confinement to the house for a day or two, with careful nursing, will cure any recent cold. And thus we might go through with the whole list of diseases and show how they might have been prevented or so controlled as not to be dangerous. Such knowledge should be as common as the ability to read and write. Any individual, and particularly one who has the care of children, is culpable who neglects to find out what it is necessary to do in any emergency that is liable to arise.

Instinct supplies this knowledge to a certain extent to the lower animals, and thus far they possess an advantage over thousands to whom human lives are intrusted.

